# Prevalence of human papillomavirus genotypes in cervical cancer in Maiduguri, Nigeria

**DOI:** 10.11604/pamj.2019.33.284.18338

**Published:** 2019-08-05

**Authors:** Abba Kabir, Mwajim Bukar, Haruna Asura Nggada, Harun Bakari Rann, Abubakar Gidado, Alhaji Bukar Musa

**Affiliations:** 1 Department of Human Pathology, University of Maiduguri, Maiduguri, Nigeria; 2 Biotechnology Centre, University of Maiduguri, Maiduguri, Nigeria; 3 Department of Mathematic and Statistics, University of Maiduguri, Maiduguri, Nigeria; 4 Department of Medical Laboratory Science, University of Maiduguri, Maiduguri, Nigeria

**Keywords:** Cervical cancer, human papillomavirus, genotypes

## Abstract

**Introduction:**

Cervical cancer is the commonest gynaecological malignancy and the second most common cancer among women worldwide. Several epidemiological, clinical and molecular studies have strongly implicated oncogenic high-risk human papillomavirus infection in the aetiopathogenesis of cervical cancer. The objectives of this study were to determine the cervical HPV prevalence and genotype distribution in cervical cancer in Maiduguri, Nigeria.

**Methods:**

This was a descriptive and retrospective study. Sixty-three archived paraffin-embedded tissue blocks with confirmed diagnoses of cervical cancer during the study period (2013-2015) were retrieved and examined. The procedure included deparaffinization of tissue samples, DNA extraction, PCR, gel electrophoresis, and HPV genotyping by reverse hybridization line probe assay.

**Results:**

Sixty-three cervical cancer cases were subjected to genomic DNA extraction and HPV-DNA detection by PCR. Fifty-eight samples showed PCR positivity while 5 samples were PCR negative. HPV-specific DNA was detected in 44 of the 58 PCR-positive samples and thus the prevalence was 69.8%. Ten different high-risk HPV genotypes were detected. Both single and multiple high-risk HPV infections were observed. The most prevalent type of the human papillomavirus detected was HPV16.

**Conclusion:**

HPV-DNA was prevalent in majority of the examined cervical cancer tissues and that HPV16, HPV18, HPV45, HPV51 and HPV52 were the predominant HPVs detected in both single and multiple HPV infections. The results of this study and further studies will provide more detailed information about HPV and may contribute significantly to the prevention of cervical cancer through primary high-risk HPV testing and HPV vaccination against the oncogenic viruses.

## Introduction

Cervical cancer is a malignant neoplasm arising from the uterine cervix-the part of the uterus connecting the body of the uterus to the vagina [1]. The burden of cervical cancer is quite high in the developing countries and constitutes a major health problem [2]. This is a source of great concern considering the fact that cervical cancer is preventable and curable at low cost with currently available methods [2]. Worldwide, it is second only to breast cancer in incidence and is the third leading cause of cancer mortality among the female population [3]. It accounts for about 8% of both total cancer cases and total cancer deaths in the world [4]. In 2012, it was estimated that there were 528,000 cases of cervical cancer, and 266,000 deaths [4]. An estimated 80% of cases of cervical cancer occur in the developing countries, and remains the second most common cancer among women in those countries [2]. In Africa, the sub-Sahara is the region with the highest incidence of cervical cancer in the world with concomitant high mortality affecting women at their prime age [2].

In Nigeria, cervical cancer is currently the second most common cancer in women after breast cancer with about 14,089 new cases diagnosed annually and 8240 deaths [5]. Most studies from different parts of Nigeria have also indicated that cervical cancer is the commonest gynaecological cancer. In Maiduguri, Northeastern Nigeria, it was the commonest gynaecological malignancy representing 70.5% of the total gynaecological cancers [6]. Molecular, clinical and epidemiological studies have shown that human papillomavirus infection plays a necessary role in the aetiopathogenesis of cervical carcinoma [3]. More than 90% of cervical carcinoma contains DNA sequences of specific HPV types [3]. A study carried out on more than 1000 invasive cervical cancer specimens at 32 hospitals in 22 different countries revealed that HPV DNA was detected in 93% of the tumours [7]. HPV-16 was present in 50%, HPV-18 in 14%, HPV-45 in 8% and HPV-31 in 5% [7]. In another study conducted to investigate regional variations in the contribution made by different HPV types to invasive cervical carcinoma, a total of 10,058 cases from 85 studies using PCR indicated that the most common HPV types in almost all the regions, in order of decreasing prevalence were: HPV-16, 18, 45, 31, 33, 58, 52, 35, 59, 56, 51, 68, 39, 82, 73, 66, and 70 [8]. The index study utilized the PCR to detect the presence of HPV-DNA in cervical cancer tissue biopsies from the University of Maiduguri Teaching Hospital. Furthermore, genotyping of the HPV-positive samples was also carried out using reverse hybridization line probe assay.

## Methods

The present study was conducted at the University of Maiduguri Teaching Hospital (UMTH) which is the apex referral centre for persons or specimens in the northeastern region of Nigeria. UMTH is located within the city of Maiduguri. This was a retrospective study of 63 cases of confirmed cervical cancer diagnosed in the Department of Histopathology of UMTH between January 2013 and December 2015. The histopathology request cards and slides were retrieved and reviewed. All the archival samples of formalin-fixed, paraffin-embedded tissues of confirmed cervical cancer within the study period were selected and subjected to the laboratory procedures. The study protocol was approved by the Research and Ethics Committee of the Hospital.

**DNA extraction:** DNA was extracted from 8-10 micron sections of FFPE tissues using *QIAamp*^®^ DNA FFPE Tissue kit from Qiagen (Hamburg, Germany) according to the manufacturer's instruction. The protocol was slightly modified to allow overnight digestion with *Proteinase K.* The tissue blocks were sectioned and processed under strict conditions to avoid potential contamination. For each block, a separate and sterile microtome blade was used after carefully and thoroughly cleaned with xylene and 70% alcohol after each cut. DNA was quantified using *NanoDrop2000C* spectrophotometer (Thermos Scientific, USA). Concentration was determined based on absorbance at 260nm. Purity was estimated as ratio of absorbance at 260nm to absorbance at 280nm (A260:A280).

**PCR for beta haemoglobin (β-globin):** PCR was run for human β-globin as a house keeping gene and to ascertain the quality of the extracted genomic DNA and the viability of the tissue for PCR detection of HPV. A primer which targets a 122-bp sequence of the β-globin was used; Forward: 5'CTTCTGACACAACTGTGTTCACTAGC 3', Reverse: 5'TCACCACAACTTCATCCACGTTCACC 3', Primers were obtained from *Inqaba Biotech West Africa.* PCR was carried out in a 25μL reaction using *One Taq Quick-load Master Mix* (New England Biolab). Reaction contain 1.25 U T*aq* polymerase in standard buffer containing 1.8mM/MgCl_2_, 22mM/NH4Cl, 22mM/KCl, 0.2μM each of forward and reverse primers and 200μM/dNTPs. To each reaction, 2μL of extracted DNA as template DNA was added. Amplification was carried out using *Mastercycler Nexus* (Eppendorf, Germany) using the following thermocycler conditions: initial denaturation at 94°C for 3min, followed by 35 cycles of denaturation at 94°C for 30secs, annealing at 56°C for 45sec and extension at 68°C for 45sec followed by final extension at 68°C for 5min.

**Gel electrophoresis:** to confirm amplification of the 122-bp β-globin, agarose gel electrophoresis was performed on 2% agarose in TAE buffer. Electrophoresis was carried out at 90V for 30min and viewed under UV trans-illuminator. A 100-kb size ladder *(Promega)* was used as the standard size DNA marker, and staining was done with Ethidium Bromide.

**Detection of HPV using *SPF10* primers:** the β-globin-positive DNA samples were subjected to PCR to detect the HPV-DNA. The SPF10 consensus primers that target a 65-base pair region of the HPV L1 open reading frame and enable the amplification of at least 54 genital HPV types were used to evaluate the presence of HPV-DNA by PCR [9]; HPV forward: 5'-GCiCAGGGiCACAATAATGG-3', HPV reverse: 5'-GTiGTATCiACAACAGTAACAAA-3'. The PCR was performed in a 25μL reaction using *One Taq Quick-load Master Mix* (New England Biolab). PCR reaction contains 1.25 U T*aq* polymerase in standard buffer containing 1.8mM/MgCl_2_, 22mM/NH4Cl, 22mM/KCl, 0.2μM each of forward and reverse primers and 200μM dNTPs. To each reaction, 2μL of extracted DNA as template DNA was added. Amplification was carried out using the following thermocycler conditions: initial denaturation at 94°C for 3min, followed by 40cycles of denaturation at 94°C for 1min, annealing at 52°C for 1min and extension at 68°C for 1min followed by final extension at 68°C for 5min.

**Gel electrophoresis:** amplification was detected using agarose gel electrophoresis. A 65-bp band was seen as positive after electrophoresis on 2% agarose in TAE buffer for 45min at 90V. Staining was done with ethidium bromide and visualized under UV trans illuminator as earlier described.

HPV genotyping

**PCR mix preparation and cycling:** PCR amplification for HPV genotyping using the INNO-LiPA HPV genotyping Extra II Amp was carried out according to the manufacturer's recommendation. Reaction was carried out in 40μL using the provided Master Mix containing biotinylated primers in buffers with dNTPs/dNTP-mix, MgCl_2_, AmpTaq Gold 360 DNA polymerase, uracil N-glucosidase and 0.05% NaN_2_ as preservative. A 10μl of HPV positive samples as detected previously was added to form a final volume of 50μL. Positive PCR control as provided in reaction kit contains HPV6 DNA and HLA-DPB DNA and 0.05 NaN_2_ as preservative. The following PCR condition was used as described by the manufacturer; 37°C,10min; 94°C, 9min [94°C, 30sec; 52°C, 450sec; 72°C, 45sec]x40. After DNA amplification, HPV genotype was determined by a reverse line probe assay for the identification of 28 different HPV genotypes. A 10μl of the purified PCR product was denatured and hybridised to the genotype-specific oligonucleotide probes immobilised as parallel lines on a nitrocellulose membrane strips, following the manufacturer's instructions (INNO-LiPA HPV genotyping kit, Innogenetics, Ghent, Belgium). Twenty-eight individual HPV genotypes can be identified simultaneously in a single assay. The result is a purple or bluish precipitate in the form of parallel lines. The 28 probes for 25 different HPV genotypes in each INNO-LiPA strip are for 18 high-risk (16, 18, 26, 31, 33, 35, 39, 45, 51, 52, 53, 56, 58, 59, 66, 68, 73, 82) and 7 low-risk (6, 11, 40, 43, 44, 54, 70) HPV types.

**Interpretation of results:** the conjugate control line on the strip was aligned to the corresponding line on the interpretation sheet, and the result of HPV genotype was read accordingly.

**Statistical data analysis:** the information on the cases of cervical cancer under review from the histopathology request cards and the results from the study were entered into a computer program. Data analysis was carried out using the statistical package for social sciences (SPSSTM) version 20.0 Chicago IL, USA, computer software. Statistical significance was assessed using the student's t-test. Correlation was evaluated using Pearson's correlation test. P value <0.05 was considered statistically significant. The number and proportions of detected HPV genotypes were determined and presented in frequency tables.

## Results

**Figure 1:**
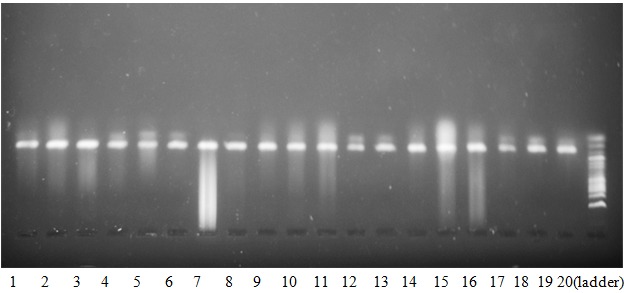
Gel electrophoresis results of amplified β-globin gene. Lanes 1-19 show a single band representing a 122-bp of the Hb beta-subunit amplified by PCR as compared to the 100-bp DNA ladder (lane 20)

**Figure 2:**
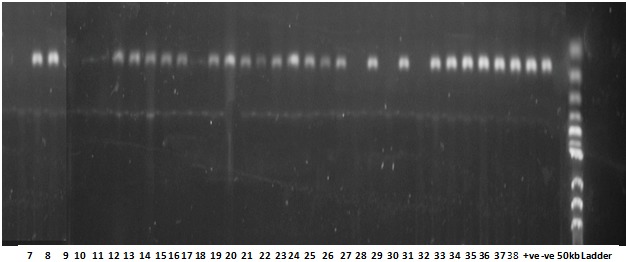
Gel electrophoresis results of some of the amplified HPV-DNA. Lanes 7, 8, 12, 13, 14, 15, 16, 18, 19, 20, 21, 22, 23, 24, 25, 26, 28, 30, 32, 33, 34, 35, 36, 37 and 38 show test samples with positive amplification showing a single band representing a 65-bp of the amplified L1 region of HPV-DNA by PCR as compared to the 50-bp DNA ladder. Lanes 9, 10, 11, 17, 27, 29 and 31 were negative for HPV L1 gene

**Prevalence of HPV-DNA in cervical cancer:** during the study period between 1^st^ January 2013 and 31^st^ December 2015, a total of 602 cases were diagnosed as cancer in the Department of Histopathology, University of Maiduguri Teaching Hospital, Maiduguri. A total of 105 (17.4%) cancer cases were of female genital tract malignancies, out of which 82 (78.1%) cases were diagnosed as cervical cancer. Sixty-three (63) cases fulfilled the inclusion criteria and were subjected to genomic DNA extraction and HPV-DNA detection by PCR. The presence and adequacy of genomic DNA extraction was confirmed by conventional PCR with human beta-globin (housekeeping) gene. Fifty-eight (58) samples showed PCR positivity while 5 samples were PCR negative. HPV-specific DNA was detected in 44 of the 58 PCR-positive samples as shown in Figure 1, Figure 2. Therefore, the prevalence of HPV-specific DNA in biopsies of cervical cancer in Maiduguri was 69.8%.

**Frequency distribution of HPV genotypes:** among the samples with a positive HPV-DNA status, ten (10) different high-risk HPV genotypes were detected. They included HPV16, HPV18, HPV31, HPV35, HPV45, HPV51, HPV52, HPV58, HPV59 and HPV73. A co-infection by low-risk HPV11 was also observed in six of the samples. Both single and multiple high-risk HPV infections were observed. There were 17 (38.6%) cases of single HPV infection and 27 (61.4%) cases of multiple HPV infections as shown in Table 1. In both the single and multiple HPV infections, the overall five most frequent high-risk HPVs detected were HPV16 (39.6%), HPV18 (19.8%), HPV45 (12.9%), HPV52 (8.9%) and HPV51 (5.0%). As a co-infection, low-risk HPV11 was observed in 6 (5.9%) cases. Together, HPV16 and HPV18 accounted for 59.4%, while the rest of the HPVs represented 40.6% (Table 1). Other less common HPVs observed were HPV31 (1.0%), HPV35 (1.9%), HPV58 (1.0%), HPV59 (2.9%) and HPV73 (1.0%) as shown in Table 1

**Table 1:** Frequency distribution of HPV genotypes in cervical cancer showing numbers and percentages of single and multiple-type HPV infections

HPV Genotype	Frequency (n)	Percent (%)
HPV16	14	31.8
HPV18	3	6.8
HPV16 & 18	2	4.5
HPV16 & 45	1	2.3
HPV16 & 52	4	9.1
HPV16 & 11	1	2.3
HPV45 & 73	1	2.3
HPV16,18 & 45	2	4.5
HPV16,18 & 52	3	6.8
HPV16,18 & 35	1	2.3
HPV16,18 & 11	1	2.3
HPV16,45 & 51	1	2.3
HPV16,31 & 52	1	2.3
HPV16,45 & 11	1	2.3
HPV16,18,45 & 51	2	4.5
HPV16,18,35 & 11	1	2.3
HPV16,18,45 & 58	1	2.3
HPV16,18,45,51 & 52	1	2.3
HPV16,18,45,59 & 11	2	4.5
HPV16,18,45,51 & 59	1	2.3
**Total**	44	100.0

HPV: Human papilloma virus

HPV 16: Single HPV infection

HPV16 & 18: Double HPV infection

HPV16,18 & 45: Triple HPV infection

HPV16,18,45 & 51: Quadruple HPV infection

HPV16,18,45,51 & 52: Quintuple HPV infection

As a single HPV infection, HPV16 and HPV18 were identified. They accounted for 14 (31.8%) and 3 (6.8%) of the total cases respectively. In multiple HPV infections, double HPV infections were observed in 9 (20.5%) cases, while triple HPV infections identified in 10 (22.7%) cases. Quadruple and quintuple HPV infections each accounted for 4 (8.2%) cases as shown in Table 1. The most commonly HPV genotypes detected in double and triple infections were HPV16 and HPV52, and HPV16, HPV18 and HPV52 respectively. In quadruple and quintuple infections, the most commonly HPVs detected were HPV16, HPV18, HPV45 and HPV51; and HPV16, HPV18, HPV45, HPV59 and HPV11 respectively (Table 1).

## Discussion

Epidemiological, clinical and molecular studies have shown that infection with high-risk HPV is the most important aetiologic agent in the pathogenesis of cervical cancer [3, 9-11]. Although there are geographical variations in the prevalence of HPV-DNA in cervical cancer tissues, worldwide, the overall prevalence has been reported to be 90-100% [10, 12, 13]. In this study, a prevalence of 69.8% of HPV-DNA in the evaluated cases of cervical cancer tissues was observed. This finding is similar to observations made in other studies conducted in Ethiopia (67.1%) [14] and Serbia (71.1%) [15]. Higher prevalence rates of HPV were reported in studies carried out in Croatia (92-7%) [11], USA (91.3%) [16], India (93.0%) [17], Thailand (94.8%) [18], Malawi (97.0%) [19], South Africa (92.1%) [20] and Ibadan Nigeria (90.7%) [21]. However, relatively low HPV prevalence rates were also observed in Poland (53.0%) [22], Khuzestan Iran (43.3%) [23] and Tehran Iran (49.0%) [24]. These differences in the reported prevalence rates of HPV in cervical cancer tissues could be attributed to several factors among which include geographical variations [10, 12, 13], quality and quantity of biological specimens [25-28], methods of DNA extraction and sensitivity [29], and specificity of HPV detection methods [27, 28, 30-32].

In this study, ten different high-risk HPV types (HPVs-16, 18, 31, 35, 45, 51, 52, 58, 59 and 73) were identified in cervical cancer cases with positive HPV-DNA status. Along with these high-risk HPVs, co-infection with low-risk HPV11 was observed in six of the analysed samples. In addition, both single and multiple HPV infections were observed. Overall, the five most prevalent high-risk HPV genotypes in this study in decreasing order of frequency were HPV16, HPV18, HPV45, HPV52 and HPV51. This finding is a global phenomenon most especially for the first three HPVs as reported in several studies across the world [10, 12, 16, 20, 33-35]. HPV16 and HPV18 were the most common HPVs detected in this study and accounted for 39.6% and 19.8% respectively and 59.4% combined. These two high-risk HPVs are the most frequently studied and consequently implicated in the causation of cervical cancer worldwide [10, 34, 36-39]. They are the most prevalent and most potent carcinogenic viruses [10, 40]. Their probability of disease progression and persistence is significantly higher than other HPVs [10]. Together, HPV16 and HPV18 account for 60-80% of the HPVs implicated in cervical cancer in the sub-Saharan Africa and most other countries in the world [10, 12, 34]. However, few studies have reported that HPV16 was not the most common HPV detected in cervical cancer due to geographical variation [25, 41]. A study by Zohoncon *et al.* [41] in Parakou, Benin Republic reported no HPV16 in cervical cancer and that HPV39 was the most prevalent genotype in their study.

A peculiar finding in this study was the detection of high-risk HPV73, which was also reported by Okolo *et al.* [21] in Ibadan Nigeria, among the studies cited above [11, 14-25]. Although it was a rare finding, it could be a Nigerian peculiarity, but more studies are needed to confirm it. An interesting observation in this study was the presence of high proportion of cases with multiple HPV infections that accounted for 61.4% of the cases with positive HPV-DNA status. A similar observation was reported in studies from two African countries-Malawi and Ghana having multiple HPV infections that accounted for 54.0% and 52.2% respectively [19, 25]. Lower rates of multiple HPV infection were reported in studies from Croatia [11], Serbia [15], India [17], Thailand [18], Ibadan Nigeria [21], Pakistan [35], Republic of Benin [41], USA [42], Brazil [43], Korea [44] and Sri Lanka [45]. Although most of the studies from Africa have observed multiple HPV infections, the finding in this study was as expected. In our environment, there is high rate of polygamous marriages; a man can marry up to four wives. In addition, divorcees, widows and widowers also remarry. In all these circumstances, the tendency of transmitting multiple HPV types is quite high. In other African countries, HIV infection, invasiveness of cervical lesions and geographical variation were suggested as factors responsible for multiple HPV infections [20, 25, 46]. The prevalence of multiple HPV infections has been attributed to multiple sexual partners, immunosuppression, smoking and pre-existing HPV [25, 41, 43, 46-48]. The significance of multiple HPV infection in cervical intraepithelial lesions and cervical cancer has conflicting reports [49]. However, most studies have identified that multiple HPV types exhibit synergism and additivity and were associated with persistent infection, higher risk of disease progression, disease severity and poor survival [17, 43, 47, 48]. Conversely, Salazar *et al.* [42] and Wang *et al.* [50] have shown no significant difference between single and multiple HPV infection, but a reduced rate of high-grade cervical lesions. Intergenotypic competition or effective immune response has been suggested as a possible mechanism for a reduced rate of high-grade cervical lesions in multiple HPV infections [42].

When taking cognizance of the available HPV vaccines (bivalent and quadrivalent HPV vaccines) and the recently introduced nonavalent vaccine (9vHPV), it can be deduced from this study that majority of HPV infections would be prevented. Bivalent and quadrivalent HPV vaccines provide protection coverage against HPV16 and HPV18; and HPV16, HPV18, HPV6 and HPV11 respectively [51, 52]. In addition, nonavalent HPV vaccine provides coverage against HPV6, HPV11, HPV16, HPV18, HPV31, HPV33, HPV45, HPV52 and HPV58 [51, 52]. In this study, the combined proportions of HPV16, HPV18, HPV31, HPV45, HPV52, HPV58 and HPV11 is 89.1%. Therefore, the bivalent, quadrivalent and nonavalent HPV vaccines would respectively cover for 59.4%, 65.3% and 89.1% of the total HPVs observed in this study. The recently introduced nonavalent HPV vaccine was granted market authorization in the USA and Europe in the mid-2015 [51]. It is considered as a safe and effective vaccine and will further reduce the incidence of HPV infection and HPV-related cancers. It can also protect unvaccinated individuals through herd immunity [51, 52].

Studies have shown the remarkable benefits of effective screening, early diagnosis and curative therapy of cervical cancer [53]. Such remarkable gains are credited to the effectiveness of the Pap test in detecting cervical precursor lesions, some of which would have progressed to cancer if not treated [53]. Despite its relatively high specificity and wide application in screening of cervical cancer, Pap smear is not very sensitive [53]. A study was conducted by Manga *et al.* [54] on comparative analysis of cervical HPV DNA testing and cytological Pap smear findings among women in tertiary health centre in northern Nigeria. It was observed that cytology had relatively high specificity but low sensitivity in detecting HPV infection, and hence suggested the introduction of HPV DNA testing to improve efficiency and maximize the sensitivity of cytology-based cervical cancer screening for women above 30 years. In another study carried out by Kolawole *et al.* [53] it was shown that high-risk HPV was present in 100% of cases of Pap smear samples with abnormal cytology, and noted that the use of HPV DNA technique would be an effective and rapid means of detecting HPV in cervical cytology specimens. Wright *et al.* [54] reported that primary high-risk HPV screening in women older than 25 years is significantly more sensitive for detection of CIN III than either cytology or hybrid strategy, although this increase is associated with more colposcopies. However, a negative result of primary high-risk HPV screening offers better reassurance of low cancer risk compared to cytology-only screening conducted at the same interval [54].

## Conclusion

This study has shown that HPV-DNA was prevalent in majority of the examined cervical cancer tissues and that HPV16, HPV18, HPV45, HPV51 and HPV52 were the predominant HPVs detected in both single and multiple HPV infections. The results of this study and further studies will provide more detailed information about HPV and may contribute significantly to the prevention of cervical cancer through the currently available HPV vaccines and HPV DNA-based screening tests against the oncogenic viruses.

### What is known about this topic

- Strong association between cervical cancer and high-risk HPVs;
- High-risk HPV types 16 and 18 are the most prevalent oncogenic viruses worldwide;
- Sensitive HPV-based tests and effective HPV vaccines have been developed.

### What this study adds

- Being the first of its kind in Maiduguri and Northeast Nigeria, the findings of this study will serve as baseline data;
- The observed high prevalence of HPV infection in cervical cancer along with the predominance of HPV16 and HPV18 is consistent with several studies worldwide;
- The high proportion of multiple HPV infections observed in this study could be explained by the high rate of polygamous marriage in our environment.

## Competing interests

The authors declare no competing interests.
